# Capacity of a Multiplex IgM Antibody Capture ELISA to Differentiate Zika and Dengue Virus Infections in Areas of Concurrent Endemic Transmission

**DOI:** 10.4269/ajtmh.20-1651

**Published:** 2021-12-20

**Authors:** Freddy A. Medina, Frances Vila, Lakshmanane Premkumar, Olga Lorenzi, Gabriela Paz-Bailey, Luisa I. Alvarado, Vanessa Rivera-Amill, Aravinda de Silva, Steve Waterman, Jorge Muñoz-Jordán

**Affiliations:** ^1^ Surveillance and Research Laboratory, Dengue Branch, Centers for Disease Control and Prevention, San Juan, Puerto Rico;; ^2^ Department of Microbiology and Immunology University of North Carolina School of Medicine, Chapel Hill, North Carolina;; ^3^ Ponce Health Sciences University, Ponce, Puerto Rico

## Abstract

Serological cross-reactivity has proved to be a challenge to diagnose Zika virus (ZIKV) infections in dengue virus (DENV) endemic countries. Confirmatory testing of ZIKV IgM positive results by plaque reduction neutralization tests (PRNTs) provides clarification in only a minority of cases because most individuals infected with ZIKV were previously exposed to DENV. The goal of this study was to evaluate the performance of a ZIKV/DENV DUO IgM antibody capture ELISA (MAC-ELISA) for discriminating between DENV and ZIKV infections in endemic regions. Our performance evaluation included acute and convalescent specimens from patients with real-time reverse transcription polymerase chain reaction (RT-PCR)-confirmed DENV or ZIKV from the Sentinel Enhanced Dengue Surveillance System in Ponce, Puerto Rico. The ZIKV/DENV DUO MAC-ELISA specificity was 100% for DENV (*N* = 127) and 98.4% for ZIKV (*N* = 275) when specimens were tested during the optimal testing window (days post-onset of illness [DPO] 6–120). The ZIKV/DENV DUO MAC-ELISA sensitivity of RT-PCR confirmed specimens reached 100% for DENV by DPO 6 and for ZIKV by DPO 9. Our new ZIKV/DENV DUO MAC-ELISA was also able to distinguish ZIKV and DENV regardless of previous DENV exposure. We conclude this novel serologic diagnostic assay can accurately discriminate ZIKV and DENV infections. This can potentially be useful considering that the more labor-intensive and expensive PRNT assay may not be an option for confirmatory diagnosis in areas that lack PRNT capacity, but experience circulation of both DENV and ZIKV.

## INTRODUCTION

Zika virus (ZIKV) and dengue virus (DENV) are flaviviruses that are transmitted through mosquito bites.^1^ Transmission mainly occurs through *Aedes aegypti* and to a smaller degree through *A. albopictus*. This results in these viruses coinciding in geographical areas within tropical and subtropical regions of the world.^2^

Zika virus gained worldwide notoriety during the 2015–2016 pandemic when the virus was observed to be a cause of congenital defects, including microcephaly in the new born.^3^ Health officials became aware of its spread in outbreaks in the Pacific in 2007. Despite serologic evidence of prior circulation of ZIKV in Africa and Asia, only a small number of cases with mild clinical symptoms were described.^4^^–^^6^ Zika virus emerged as a new American subclade from the Asian lineage in the Americas and the Caribbean. The lack of immunity throughout the population facilitated its dissemination.^7^

Dengue viruses exist as four serotypes (DENV-1–4) that share a ∼55% amino acid sequence similarity with ZIKV.^8^ Individuals can be infected with each one of the four DENVs.^9^ It is estimated that approximately 400 million people could be infected every year.^10^ The number of DENV cases in the Americas continues to grow steadily, reaching a record of over 3 million cases in 2019.^11^

Zika virus and DENV are very similar. They are single-stranded positive sense RNA viruses of ∼11 kb. Their genomes encode a single polyprotein that is cleaved into structural (C, prM, and E) proteins that form the infectious virion containing the viral genome and nonstructural (NS1–NS5) proteins that are involved in virus replication.^12^ During the acute phase of illness, both viruses present with similar clinical features, highlighting the need for accurate diagnostic tests.^13^ The most reliable tests for distinguishing these viruses consist of nucleic acid detection in serum for DENV and serum, urine, and saliva for ZIKV.^5^^,^^14^^,^^15^ However, there are circumstances under which testing does not occur in a timely manner to permit molecular testing during an optimal testing window. Some examples are patients delaying seeking medical care or serum sample submission, delays in medical referral, and travelers returning from an endemic country. Interest in distinguishing these viruses may come from pregnant women with concerns of potential ZIKV fetal exposure or men infected with ZIKV because they could have potentially transmitted the virus sexually to their partner. Therefore, there is a need to use serological methods during periods of increased ZIKV circulation in endemic areas to indirectly determine the infecting virus when molecular testing is no longer reliable.

Plaque reduction neutralization tests (PRNTs) are common to identify the infecting virus in flavivirus IgM positive cases, but they have limitations.^16^^,^^17^ Individuals experiencing their first flavivirus infection can be correctly diagnosed by PRNT most of the time.^18^ However, in regions where two or more flaviviruses can occur, it may be challenging to determine the infecting virus near the acute phase.^19^ The presence of flavivirus IgG from prior infection and the development of IgG soon after the development of IgM prevents a definitive diagnosis because of the cross-neutralization of noninfecting viruses in PRNTs. Recent studies have shown that the PRNTs may not confirm some IgM positive cases in the continental United States, or fail to differentiate ZIKV and DENV infections in Puerto Rico, where the population has been exposed to both viruses.^20^ Moreover, PRNTs are laborious, low throughput, costly, and need to be performed by skilled technicians in highly specialized laboratories. The most practical alternative to a PRNT would be using an ELISA assay, but the current level of flavivirus cross-reactivity is too high for an accurate diagnosis. The temporary rise in IgM and with the higher reactivity to the antigen of the infecting virus permit the development of methods that can minimize the effect of flavivirus IgM cross-reactivity.

Here we present data on the performance of a MAC-ELISA (Z/D DUO ELISA) that was developed to differentiate between recent infections of ZIKV and DENV. We used specimens from well-characterized cases that were real-time reverse transcription polymerase chain reaction (RT-PCR) positive for ZIKV or DENV. A receiver operating characteristic (ROC) analysis was performed to determine the assay cutoff and the assay was then evaluated with specimens collected at different time intervals to determine the optimal testing window of the assay. Specimens from West Nile virus (WNV) and Yellow Fever virus (YFV) were also tested to assess their cross-reactivity in the assay. The ZIKV/DENV DUO MAC-ELISA was also evaluated during an outbreak investigation of DENV in American Samoa approximately 6 months after the ZIKV outbreak ended.^21^ Our results demonstrate that the ZIKV/DENV DUO MAC-ELISA reaches a sensitivity of 100% for ZIKV or DENV compared with RT-PCR in the acute phase and approximately 90% compared with the CDC ZIKV MAC-ELISA and 96% compared with the InBios DENV Detect IgM Capture ELISA when tested in the days post-onset of illness (DPO) 6–120 window. This ELISA is meant to be used in areas where there is possible co-circulation and can be used in resource-constrained regions as a result of the low cost of implementation.

## MATERIALS AND METHODS

### Specimen collection.

Written informed consent or assent was obtained from all study participants according to a protocol with ethics approval by the CDC and Ponce Medical School Foundation, Inc (PMSF) Institutional Review Board for sample collection and use. The CDC, together with the PMSF, established the Sentinel Enhanced Dengue and Acute Febrile Illness Surveillance System (SEDSS) in 2012 to improve the understanding of the incidence, etiology, and clinical course of febrile patients presenting for care at two hospitals in southern Puerto Rico. Participants are identified during triage when they present with acute febrile illness (AFI) defined by presence of fever (≥ 38.0°C or 100.5°F) or report having a fever lasting 7 days or less. Since only a subset of DENV and ZIKV cases present with fever, eligibility criteria for SEDSS also includes rash, arthralgia/arthritis, or conjunctivitis. All eligible participants are invited to participate in SEDSS. As part of SEDSS, physicians order blood, nasopharyngeal swabs, and urine to be collected for all participants. Specimens are sent for testing at the CDC Dengue Branch Laboratory in San Juan, Puerto Rico, where testing is done for DENV, ZIKV, chikungunya virus (CHIKV), and other respiratory infectious diseases.

For random sample selection, a new unique identifier was generated using the NEWID function in MySQL and specimens where then organized from lowest to highest number based on the new identifier assigned.

The WNV and YFV serum samples are part of a preexisting de-identified collection at the CDC Arboviral Diseases Branch, and thus considered exempt from human subject research.

### Receiver operating characteristic analysis and serum immunology.

GraphPad Prism 8.0 (GraphPad Software Inc., San Diego, CA) was used to plot sensitivity against 100-specificity to obtain the ROC curve of RT-PCR confirmed ZIKV, DENV, and negative specimens to evaluate optimal thresholds for differentiating the viruses and individuals not recently infected and to calculate the area under curve. This allowed us to determine the ratio for separating ZIKV from DENV and negative specimens. Calculations for distinguishing DENV positive from negative specimens were performed using positive-to-negative absorbance ratios as previously described.^16^ Our validation set of specimens for the establishment of the assay cutoff included DPOs 1–36. Based upon the results obtained with the training set samples, an equivocal zone was established where specimens with values falling within this range could not be reliably determined even with repeat testing.

#### Virus-like particle production.

Procedures for the construction and expression of virus-like particles (VLPs) from DENV-2 and DENV-1, 3, and 4 were described previously.^22^^,^^23^ Virus-like particles for ZIKV were generated similarly with the exception that the premembrane/envelope (prM/E) of ZIKV BPH-2016 strain (Brazil 2016) was codon optimized for improved transcription and translation and cloned into a eukaryotic cell expression plasmid. Stable cell lines were generated and tissue-culture medium containing ZIKV VLPs, DENV-1–4 VLPs, or negative control COS-1 cells were harvested and clarified by centrifugation to obtain antigens used for the MAC-ELISA.

#### ZIKV/DENV DUO MAC-ELISA.

Immulon 2HB microtiter plates (Thermo Scientific, Rochester, NY) were coated with 75  µL of goat antihuman IgM (Kirkegaard and Perry Laboratories, Gaithersburg, MD) diluted 1:2,000 in coating buffer (Carbonate/bicarbonate buffer, pH 9.6. 1.59 g Na_2_CO_3_ +2.39 g NaHCO_3_ diluted 1 L water) and incubated overnight at 4°C. The capture antibody was dumped, and wells were blocked by incubation at room temperature for 30 minutes with blocking buffer (5% Nonfat Dry Milk/0.5% Tween-20 in FTA Hemagglutination Buffer). Plates were washed five times with wash buffer (FTA/0.05% Tween-20). Normal human serum negative controls and DENV and ZIKV positive controls from previously characterized specimens were included in every plate. Sera and controls were tested in duplicate for each antigen and diluted (1:100) in wash buffer, incubated for 1  hours at 37°C in a humid chamber, and washed five times. Dengue virus-1–4 COS-1 produced VLPs, ZIKV COS-1 produced VLPs or COS-1 supernatant normal cell antigen (NCA) as a negative control were added at determined optimal dilutions in high salt concentration 1X PBS (500 mM NaCl, 2.7 mM KCl, 1.0 mM KH2PO_4_, 6.4 mM Na_2_HPO_4_, pH 7.4) and added to each serum specimen in duplicate and incubated 1 hour at 37°C in a humid chamber. Horseradish peroxidase conjugated pan-flaviviral monoclonal antibody 6B6C-1 diluted 1:5,000 in blocking buffer was added to each well and incubated for 1 hour at 37°C in humid chamber. After washing 10 times (changing plate position after five washes), Neogen TMB substrate was added, plates were incubated at room temperature in the dark for 10 minutes, and the reaction stopped with 1N H_2_SO_4_. The absorbance was measured at 450 nm with and without a blank.

#### Ratio calculations.

The Z/D ratio (using data without a blank) = (Mean optical density [OD] of Zika Ag/Mean OD of Dengue Ag) for controls and test samples.

$$\frac{\begin{array}{c} \text{Mean}\;\text{OD}\;\text{of}\;\text{the}\;\text{serum}\;\text{specimen}\;\text{reacted}\;\text{with}\\ \text{ZIKV}\;\text{COS}-\text{1}\;\text{produced}\;\text{VLP}\;\text{antigen}\left(\text{Z}\right) \end{array}}{\begin{array}{c} \text{Mean}\;\text{OD}\;\text{of}\;\text{the}\;\text{serum}\;\text{specimen}\;\text{reacted}\;\text{with}\\ \text{DENV}\;\text{COS}-\text{1}\;\text{produced}\;\text{VLP}\;\text{antigen}\;\left(\text{D}\right) \end{array}}$$

Specimens with a Z/D ratio greater than or equal to 2.0 are classified as ZIKV IgM positive. Those with ratio values of 1.70–1.99 are considered equivocal for ZIKV and not further tested. All specimens with ratios of 1.69 and lower are then evaluated with the DENV IgM ratio using blanked data.

$$\frac{\begin{array}{c} \text{Mean}\;\text{OD}\;\text{of}\;\text{the}\;\text{serum}\;\text{specimen}\;\text{reacted}\;\text{with}\;\\ \text{DENV}\;\text{COS}-\text{1}\;\text{produced}\;\text{VLP}\;\text{antigen}\left(\text{D}\right) \end{array}}{\begin{array}{c} \text{Mean}\;\text{OD}\;\text{of}\;\text{the}\;\text{normal}\;\text{human}\;\text{control}\;\text{serum}\;\text{reacted}\;\text{with}\\ \text{DENV}\;\text{COS}-\text{1}\;\text{produced}\;\text{VLP}\;\text{antigen}\;\text{Calibrator}\;\text{Control}\;\left(\text{CC}\right) \end{array}}$$

Specimens with a D/CC ratio greater than or equal to 3.0 are classified as DENV IgM positive. Those with ratio values of 2.0–2.99 are considered equivocal for DENV and those with a ratio below 2.0 are considered negative for ZIKV and DENV.

All positive specimens are evaluated for nonspecific background reactivity. The mean OD of the serum specimen reacted with DENV or ZIKV VLP antigen for the test specimen must be greater than or equal to twice (2X) the mean OD of the test specimen reacted with NCA. Those that do not fulfill the criteria are considered inconclusive.

#### ZIKV EDIII IgG ELISA.

The ZIKV EDIII IgG ELISA is a modified version from a previously published report.^24^ Plates were coated with streptavidin at a concentration of 4 µg/mL in coating buffer 1X TBS (25 mM Tris-HCl, 137 mM NaCl, 2.7 mM KCl, pH 7.4) and incubated at 37°C for 1 hour. After three washes, wells were blocked with blocking solution (3% Nonfat Dry Milk/0.05% Tween-20 in 1X TBS) at 37°C for 1 hour. The blocking solution was discarded, and biotinylated EDIII antigen diluted to 2 µg/mL in blocking solution was added for a 1 hour incubation at 37°C. Plates were washed three times and serum diluted (1:20) in blocking solution. Plates were incubated for 1 hour at 37°C and washed three times with wash buffer. Fresh antihuman IgG alkaline phosphatase (AP) conjugate (Sigma-Aldrich, St. Louis, MO) diluted in blocking solution (1:2,500) was added and incubated for 1 hour at 37°C. Plates were washed three times, SigmaFast AP substrate was added for 15 minutes at room temperature and plates read on a plate reader at the 405 nM wavelength setting.

#### CDC ZIKV MAC-ELISA and InBios DENV detect IgM capture ELISA.

The CDC ZIKV MAC-ELISA was performed as previously described by Basile et al.^25^ Testing by InBios DENV Detect IgM Capture ELISA was performed according to manufacturer’s instructions.

### Statistical analysis.

Student’s *t*-test was used for comparisons between normally distributed continuous variables. All statistical analyses were performed using GraphPad Prism version 6.0 (GraphPad Software, San Diego, CA), and significance level was set at a *P* value < 0.05.

## RESULTS

**Figure 1.**
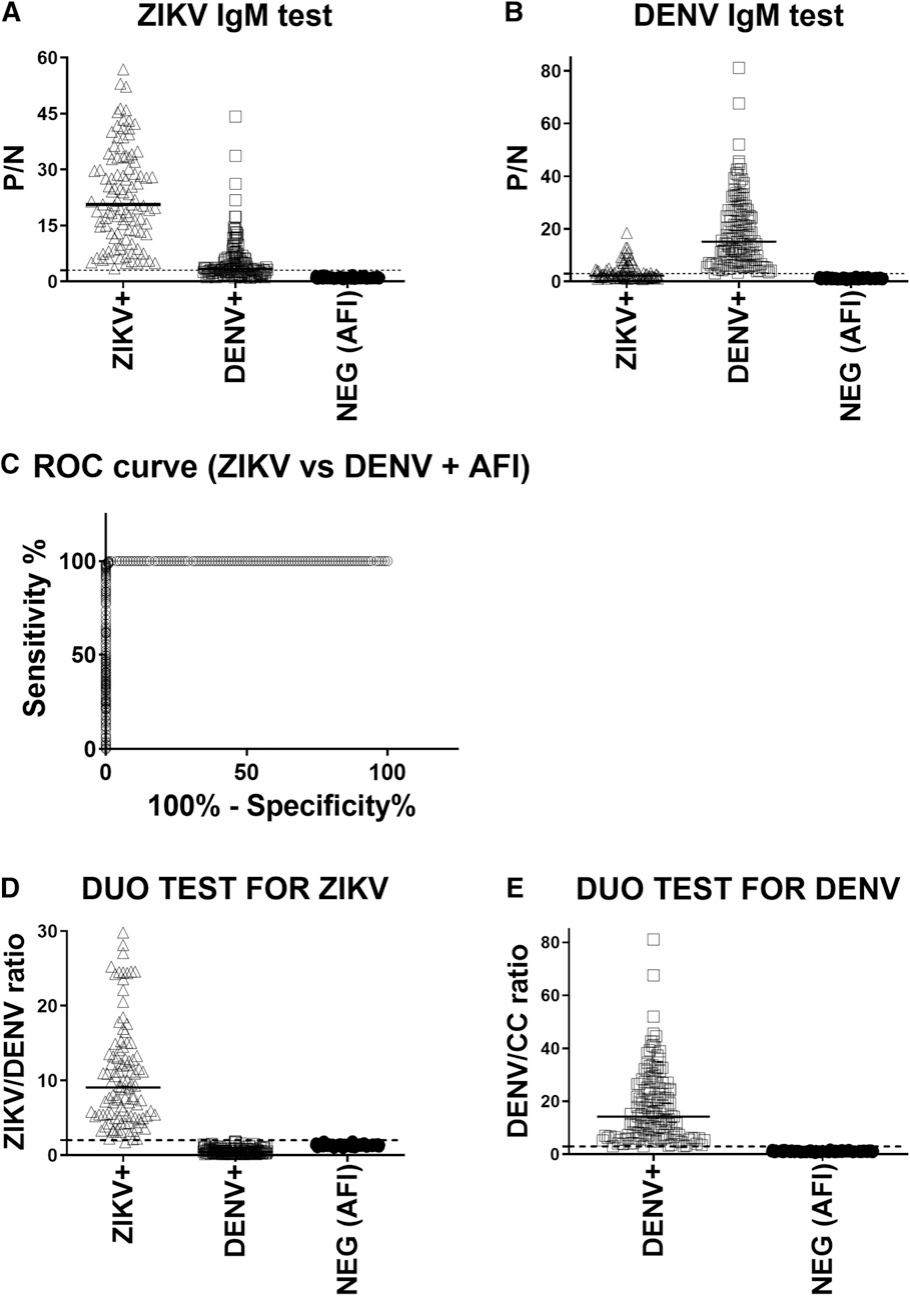
The ZIKV/DENV DUO MAC-ELISA can discriminate ZIKV and DENV infections. Specimens from RT-PCR confirmed ZIKV (*N* = 103) cases, DENV (*N* = 134) cases, and negative (*N* = 143) cases were tested by (**A**) ZIKV and (**B**) DENV IgM MAC-ELISAs. (**C**) Represents the ROC curve for the detection of ZIKV or non-ZIKV (DENV and other acute febrile illnesses) specimens using the ZIKV/DENV ratio. (**D**) Differentiation of ZIKV from DENV and negative specimens using the ZIKV/DENV ratio for the ZIKV DUO test. (**E**) Differentiation of DENV and negative specimens using the DENV/CC ratio for the DENV DUO test. **P  <  0.0001 (*P* values were calculated using a two-tailed Student *t*-test). Statistical significance is indicated with asterisk (**P < 0.01). Unpaired two-tail *t* test was used for comparison among groups. #Indicates a statistically significant difference between the study groups (*P* < 0.05).

The ZIKV/DENV DUO MAC-ELISA cutoff was established using characterized convalescent specimens as a training set. These specimens were first tested using IgM Antibody Capture ELISAs, also known as MAC-ELISAs, to determine the amount of cross-reactivity observed in these tests because they are traditionally performed in the diagnosis of flavivirus infections using serology. We tested ZIKV (*N* = 103), DENV (*N* = 134), and negative (*N* = 143) specimens collected at our clinical site, SEDSS. Zika virus specimens used were collected during the 2016–2017 ZIKV epidemic in Puerto Rico and DENV specimens were collected before the ZIKV epidemic from 2012 to 2014. All negative specimens selected were negative in the CDC Trioplex RT-PCR and IgM negative in the convalescent specimen for DENV, ZIKV, and CHIKV. The convalescent specimens of negative cases were used in the evaluation. All ZIKV specimens (103/103 = 100%) tested positive in the ZIKV IgM test and no negative specimens (0/143 = 0%) tested positive. The ZIKV specimens displayed significant cross-reactivity (35/103 = 34%) in the DENV IgM test. All DENV specimens tested in the DENV IgM tested were all positive (142/142 = 100%) and negative specimens (0/143 = 0%) tested negative. A high proportion of DENV specimens (78/134 = 58%) were cross-reactive in the ZIKV IgM assay (Figure 1A). While IgM cross-reactivity occurs after recovery from DENV or ZIKV infection, the frequency of cross-reactivity was greater after recovery from DENV infection than with ZIKV infection (Figure 1B). Pairwise comparisons (adjusted for multiple comparisons by the Bonferroni method) revealed that there was a significant difference in correctly identifying ZIKV and negative cases compared with DENV cases in the ZIKV IgM ELISA (Supplemental Table 1A). Similarly, there were significant differences in correctly identifying DENV and negative cases compared with ZIKV cases in the DENV IgM ELISA (Supplemental Table 1B). This demonstrates that DENV cases cannot be reliably identified in the ZIKV IgM ELISA and ZIKV cases cannot be reliably identified in the DENV IgM ELISA.

Convalescent specimens from individuals who experience DENV and ZIKV have a higher reactivity to their homotypic antigen. That is, ZIKV specimens have higher OD values to ZIKV antigen than DENV antigen and DENV specimens have higher OD values to DENV antigen than ZIKV antigen. We used this to determine the OD ratio to differentiate ZIKV specimens from DENV and other negative specimens from AFI. Then DENV and negative specimens from other febrile illnesses are distinguished with the traditional calculations used in flavivirus MAC-ELISAs.^17^

The ROC curve, shown in Figure 1C, was generated by plotting the sensitivity of the test against (100—specificity) using RT-PCR classification as the gold standard. The area under the ROC curve was found to be 0.9999, which indicates the highest level of test accuracy. Differentiation of ZIKV from DENV and negative specimens of other AFI was achieved using the optimal cutoff that was determined to be a value of 2.

Testing of ZIKV specimens in the ZIKV/DENV DUO ELISA showed that (102/103 = 99%) of them were correctly identified as positive for ZIKV IgM (Figure 1D). Dengue virus and AFI specimens evaluated in the ZIKV DUO portion of the ZIKV/DENV DUO ELISA were mostly negative. There were (4/134 = 3%) of DENV specimens and (2/143 = 1.4%) of AFI specimens that tested equivocal for ZIKV. No further testing of ZIKV equivocal specimens is done because retesting was shown to be not reliable. Zika virus cases were shown to be reliably distinguished from DENV and negatives. Since the utilization of the ZIKV/DENV ratio identifies ZIKV specimens and excludes all other infections, the remaining specimens are then calculated under the DENV/CC ratio to determine specimens that have DENV IgM present (Figure 1E). Of the remaining DENV specimens (130/130 = 100%) were positive for DENV IgM and no AFI specimens (0/141 = 0%). Dengue virus and negative cases were shown to be reliably distinguished equally from ZIKV cases in pairwise comparisons (adjusted for multiple comparisons by the Bonferroni method). The proportion of DENV and negative specimens correctly distinguished from ZIKV was not statistically different (Supplemental Table 1C). All DENV cases were shown to be distinguished from negative cases and therefore could not be compared statistically (Supplemental Table 1D).

**Figure 2.**
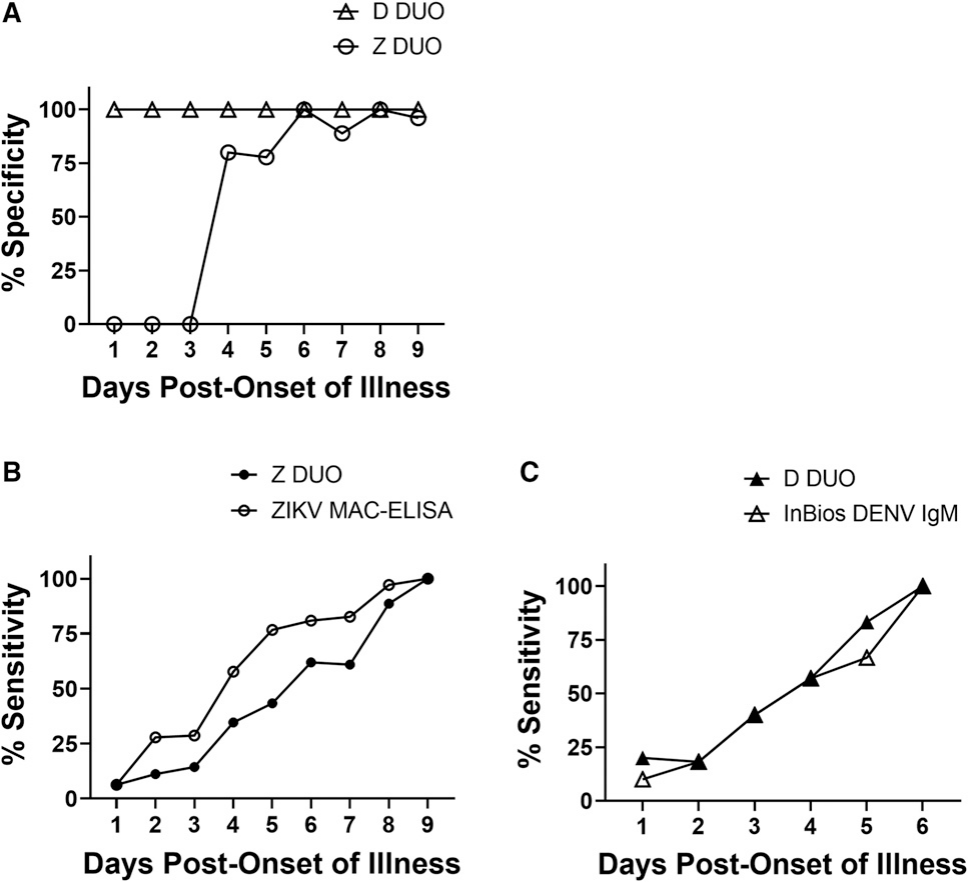
(**A**) Specificity and sensitivity (**B** and **C**) of the ZIKV/DENV DUO ELISA for ZIKV (*N* = 216) or DENV (*N* = 86) infections during the acute phase. RT-PCR confirmed specimens were randomly selected at a range of days post-onset of illness (DPO) for ZIKV (DPO 1–9) or DENV (DPO 1–6). The specificity was determined by comparing the ZIKV/DENV DUO ELISA interpretation with the RT-PCR result. The sensitivity of the assay was compared with the (**B**) CDC ZIKA MAC-ELISA or (**C**) the InBios DENV Detect IgM.

The established ZIKV/DENV cutoff was evaluated using DENV and ZIKV RT-PCR confirmed specimens collected at SEDSS and selected randomly from DPO 1–9 to determine the optimal window for testing. The samples for DPO 1–6 were RT-PCR positive and samples in DPO 7–9 had a paired acute sample that tested RT-PCR positive. This was done to improve reliability of DPO assignment reported by patients. Results showed that DENV specimens were correctly identified 100% of the time. ZIKV specimens were not reliably identified until DPO 6 (Figure 2A). The sensitivity of the ZIKV/DENV DUO ELISA was lower than the CDC ZIKV MAC-ELISA, but by DPO 9 all ZIKV specimens had detectable IgM (Figure 2B). When compared with the United States Food and Drug Administration (FDA) cleared InBios DENV Detect IgM Capture ELISA, the sensitivity of tests was equivalent during the acute stage of infection (Figure 2C). We further analyzed the ZIKV/DENV DUO ELISA performance under several testing windows ranging from DPO 4–120 to DPO 9–120. Testing for ZIKV and DENV IgM achieved at least 90% sensitivity and specificity for both pathogens by DPO 6 (Table 1). Therefore, as a result of these results and those observed during testing in the acute stage we established our testing window for the assay to be DPO 6–120. There was no difference in test performance between cases experiencing a primary ZIKV infection and those who had multiflavivirus infections or DENV infection before ZIKV (Table 2).

**Table 1:** ZIKV/DENV DUO ELISA performance by sliding windows of DPO

DPO	4–120	5–120	6–120	7–120	8–120	9–120	Virus
Specificity*	100%	100%	100%	100%	100%	100%	DENV
	(*N* = 190)	(*N* = 159)	(*N* = 127)	(*N* = 104)	(*N* = 75)	(*N* = 60)	
Sensitivity†	98.3%	98.7%	96.1%	95.2%	94.7%	93.3%	DENV
	(*N* = 190)	(*N* = 159)	(*N* = 127)	(*N* = 104)	(*N* = 75)	(*N* = 60)	
Specificity*	97.1%	97.3%	98.4%	98.3%	99.5%	99.3%	ZIKV
	(*N* = 356)	(*N* = 312)	(*N* = 275)	(*N* = 251)	(*N* = 206)	(*N* = 166)	
Sensitivity†	82.9%	86.4%	90.4%	91.6%	91.2%	92.2%	ZIKV
	(*N* = 356)	(*N* = 312)	(*N* = 275)	(*N* = 251)	(*N* = 206)	(*N* = 166)	

DENV = dengue virus; DPO = days post-onset of illness; ZIKV = Zika virus.

* Calculated from any IgM positive specimen classified by RT-PCR.

† Sensitivity compared with CDC ZIKV MAC-ELISA. Equivocals were considered negative for calculations.

**Table 2:** Sensitivity and specificity for convalescent (DPO 6–120) primary and multiflavi exposed ZIKV specimens tested in the ZIKV/DENV DUO MAC-ELISA

Immune status	Specificity (%)	Sensitivity (%)
Primary ZIKV	100	94.3
Multiflavi	98.2	91.1

DENV = dengue virus; DPO = days post-onset of illness; ZIKV = Zika virus.

We randomly selected specimens collected longitudinally between DPO 6 and 705 that were ZIKV IgM positive in the CDC ZIKV MAC-ELISA to evaluate the performance of the ZIKV/DENV DUO ELISA as a tool for screening women for possible ZIKV exposure during pregnancy. Zika virus specimens of nonpregnant and pregnant cases tested between DPO 6 and 120 had an overall specificity of 100%. A decrease in specificity to 83.3% was observed in specimens with DPO over 120. The average DPO in these specimens was 252 and the furthest DPO was of 705. The average sensitivity of ZIKV nonpregnant and pregnant cases tested between DPO 6 and 120 was 86.2%. A higher sensitivity was observed in specimens from nonpregnant (89.7%) than pregnant (76.9%) women despite no significant difference in the average DPO between the groups. Overall, ZIKV specimens tested at DPO over 120 had decreased specificity (83.3%) compared with those tested between DPO 6 and 120. The specificity in specimens from nonpregnant cases was 80% and for pregnant cases 100%. Test sensitivity was very low (26.7%) in ZIKV specimens above DPO 120. For specimens from nonpregnant cases it was 38.5% and 10.5% for pregnant cases (Table 3).

**Table 3:** Sensitivity and specificity of ZIKV specimens from the ZIKV study tested in the ZIKV/DENV DUO MAC-ELISA

	All ZIKV		Nonpregnant		Pregnant		
	Specificity*	Sensitivity†	Specificity*	Sensitivity†	Specificity*	Sensitivity†	Virus
Acute convalescent (DPO 6–120)	100%	86.2%	100%	89.7%	100%	76.9%	ZIKV
	(*N* = 81)	(*N* = 94)	(*N* = 61)	(*N* = 68)	(*N* = 20)	(*N* = 26)	
Remote (DPO > 120)	83.3%	26.7%	80%	38.5%	100%	10.5%	ZIKV
	(*N* = 12)	(*N* = 45)	(*N* = 10)	(*N* = 26)	(*N* = 2)	(*N* = 19)	

DENV = dengue virus; DPO = days post-onset of illness; ZIKV = Zika virus.

* Calculated from IgM positive specimens.

† Sensitivity compared with CDC ZIKV MAC-ELISA. Equivocals were considered negative for calculations.

**Table 4:** Low cross-reactivity of West Nile virus specimens tested in the ZIKV/DENV DUO ELISA

Specimen	DUO ELISA result	DPO	PRNT titer
WN1	Equivocal for DENV	5	320
WN2	Negative	30	160
WN3	DENV positive	48	20,480
WN4	Equivocal for ZIKA	8	320
WN5	Equivocal FOR DENV	8	1,280
WN6	Equivocal for DENV	80	640
WN7	Negative	27	1,280
WN8	Equivocal FOR ZIKV	2	1,280
WN9	Negative	10	320
WN10	Negative	47	160

DENV = dengue virus; PRNT = plaque reduction neutralization test; ZIKV = Zika virus.

**Table 5:** Low cross-reactivity of Yellow fever virus vaccinated specimens tested in the ZIKV/DENV DUO ELISA

Specimen	DUO ELISA result	YF MAC-ELISA
YF1	Negative	Positive
YF2	Negative	Positive
YF3	Equivocal for DENV	Positive
YF4	Negative	Positive
YF5	Negative	Positive
YF6	Negative	Positive

DENV = dengue virus; ZIKV = Zika virus.

**Table 6:** Testing of specimens from a DENV-2 outbreak in American Samoa after a recent ZIKV outbreak in the ZIKV/DENV DUO ELISA

DUO ELISA RESULT	ZIKV IgG negative	ZIKV IgG positive	Total
Negative	20/36 (55.5%)	14/29 (48%)	34/65 (52%)
DENV positive	11/36 (30.5%)	8/29 (28%)	19/65 (29%)*
ZIKV positive	0/36 (0%)	3/29 (10.3%)	3/65 (5%)
Equivocal for DENV	5/36 (14%)	1/29 (3.4%)	6/65 (9%)
Equivocal for ZIKV	0/36 (0%)	3/29 (10.3%)	3/65 (5%)

DENV = dengue virus; ZIKV = Zika virus.

* 14/65 (22%) specimens were positive in the InBios DENV Detect IgM.

To examine possible cross-reactivity of other flaviviruses in the ZIKV/DENV DUO ELISA we tested serum specimens from WNV infected cases and YFV vaccinated individuals. Most WNV specimens (9/10) did not test positive for ZIKV or DENV (Table 4). There were four negatives, two equivocal for ZIKV, three equivocal for DENV, and one tested DENV positive. The DENV positive specimen also had the highest WNV PRNT endpoint titer. For YFV specimens, 5/6 tested negative for both ZIKV and DENV and one specimen was equivocal for DENV (Table 5).

To have a better understanding of how the ZIKV/DENV DUO ELISA would perform in a scenario of a DENV outbreak after ZIKV, we tested specimens from the 2016–2018 DENV-2 outbreak in American Samoa. The last ZIKV RT-PCR positive case detected in American Samoa was in June 2016 and the first DENV cases were detected in November 2016. We tested a total of 65 specimens with enough volume that were DENV RT-PCR or DENV NS1 Ag positive collected during the epidemic by an anti-ZIKV EDIII IgG ELISA to determine previous exposure to ZIKV and in the ZIKV/DENV DUO ELISA. All specimens were tested in the acute phase (DPO 1–5) and outside of the optimal testing window for ZIKV/DENV DUO ELISA (DPO 6–120). No paired specimens were available. We did not detect IgM in almost half (52%) of the cases. There were 19 specimens positive for DENV IgM and three specimens positive for ZIKV IgM in the ZIKV/DENV DUO ELISA. Of the 65 specimens tested 14 were positive in the InBios DENV Detect IgM. Specimens that were negative for ZIKV IgG were either negative for both ZIKV and DENV IgM or positive or equivocal for DENV IgM only. There were 29 specimens from individuals that likely had prior ZIKV exposure. No IgM for DENV or ZIKV was detected in 48% of these specimens, 28% were positive for DENV IgM only, 10.3% for ZIKV IgM only, 3.4% equivocal for DENV, and 10.3% equivocal for ZIKV (Table 6).

## DISCUSSION

The spread of ZIKV throughout DENV endemic areas has brought about challenges in diagnostic serology. The ZIKV/DENV DUO ELISA was developed based upon the hypothesis that confirmation and discrimination of recent infections is more likely to occur by the detection of IgM, an isotype that is detectable early in infection and then wanes over time, than by the detection of neutralizing antibodies that are more likely to be reflective of both recent and remote infections. Conventional testing of flaviviruses involves the detection of IgM by MAC-ELISA, followed by confirmation by PRNT. Studies have shown that the use of PRNT during the acute phase is unreliable, and the cross-reactivity generated in MAC-ELISAs causes false positive and inconsistent results.^20^ The data presented here corroborate that 58% of DENV specimens are positive in a ZIKV IgM test, and 34% of ZIKV specimens were positive in a DENV IgM test. Studies have shown that IgM reactivity is lower against other flaviviruses the individual is not infected with.^16^ We used this knowledge to leverage our ability to differentiate ZIKV and DENV infections by IgM serology using the OD ratio of the samples reacting against both antigens. In addition, our ELISA utilizes a higher salt concentration than what is used in traditional flavivirus MAC-ELISAs, 500 mM NaCl compared with 137 mM NaCl, which reduces the binding of nonspecific DENV IgM antibodies to ZIKV antigen (Supplemental Table 2). DENV specimens tested in the ZIKV/DENV DUO MAC-ELISA were detected at a similar rate to the FDA-cleared InBios DENV Detect IgM Capture ELISA. Detection of ZIKV specimens in the ZIKV/DENV DUO ELISA was lower compared with the CDC DENV ZIKV MAC-ELISA; however, detection of IgM in 100% of RT-PCR positive cases was achieved by DPO 9. The lower sensitivity between the two assays was expected because the ZIKV/DENV DUO ELISA was designed as a 1-day assay for use in high throughput laboratories and the CDC DENV ZIKV MAC-ELISA is a 2-day assay designed for maximum IgM sensitivity. In the present study, testing on specimens collected   ≥  DPO 6 and up to DPO 120 revealed a 98.4% specificity for ZIKV and 100% specificity DENV specimens in the ZIKV/DENV DUO ELISA. The sensitivity for ZIKV specimens was 90.4% and for DENV 96.1%. Testing of ZIKV specimens over DPO 120 was unreliable and showed both a decrease in specificity to 83.3% as well as sensitivity to 26.7%. Therefore, testing of pregnant women every trimester as recommended by CDC during periods of high ZIKV circulation is a possibility with the high test accuracy observed up to DPO 120. More importantly, the ZIKV/DENV DUO ELISA performed well regardless of prior exposure to DENV in ZIKV cases and had minimal cross-reactivity to WNV and YFV samples tested in the assay. The sole WNV specimen that tested positive for DENV had very high anti-WNV neutralizing antibodies levels. Despite the minimal cross-reactivity in the ZIKV/DENV DUO ELISA, areas with potential WNV and YVF co-circulation should also consider devising serological diagnostic algorithms based on geographical location to differentiate flaviviruses if active transmission is confirmed by molecular tests.

The evaluation of the DENV/ZIKV DUO ELISA with DENV-2 specimens collected approximately 6 months after a ZIKV outbreak demonstrated the utility of the assay despite testing the specimens outside of the ideal DPO 6–120 testing window. The specimens were collected at an average DPO of 2.The first ZIKV RT-PCR positive case for ZIKV detected in American Samoa was in January 2016 and the last RT-PCR positive case was detected in June 2016.^26^ Dengue virus was first detected in November 2016, circulation peaked in December 2017 and ended in October 2018.^21^ The ZIKV/DENV DUO ELISA detected DENV IgM in 19/65 (29%) compared with 14/65 (22%) for InBios DENV Detect IgM. This demonstrates that both assays display similar sensitivity. All American Samoa specimens that did not have prior ZIKV exposure only tested positive for DENV IgM in the ZIKV/DENV DUO ELISA. There were three DENV RT-PCR positive specimens that were ZIKV IgM positive. Since these specimens were tested outside the optimal testing window and they also tested ZIKV IgG positive, suggesting prior ZIKV exposure, the ZIKV/DENV DUO ELISA result alone would not be reliable. It has been shown that the duration of ZIKV IgM can occur for over a year after exposure; therefore, the ZIKV IgM detected is most likely from a prior infection that occurred during the ZIKV outbreak in American Samoa.^27^ Since it takes between 8 and 9 days for ZIKV IgM to develop in 100% of cases, an additional sample taken at that time could increase the likelihood of obtaining a correct diagnosis.

Our study is not without limitations. The collection of samples occurred during different periods. Dengue virus cases were obtained during the period 2012–2014, before ZIKV arrival and the ZIKV and other AFI specimens were obtained in 2016–2017. Zika virus cases were sampled longitudinally up to 18 months, whereas DENV cases were not followed longitudinally. Therefore, we did not have confirmed DENV specimens over DPO 120 for testing. Although we could compare our results to a gold standard classification of RT-PCR, we do not have a side-by-side comparison to compare the assay accuracy to PRNT.

There are several reports of diagnostic tests for differentiating DENV and ZIKV. Some strategies involved mutating the fusion loop domain of the envelope protein.^28^ Others used NS1 protein as the antigen rather than the envelope protein for increased specificity, but the duration of detection of anti-NS1 antibodies is more limited.^29^ The use of a monoclonal anti-ZIKV NS1-antibody was used to develop a Zika NS1 blockade-of-binding ELISA to discriminate ZIKV infection from other flaviviruses.^30^ The serological diagnosis of ZIKV was done using a microarray-based assay with ZIKV NS1 as antigen and DENV virus particles in a multiplexed format based on a plasmonic gold platform.^31^ The use of our assay allows a simple, low cost, and high throughput approach for differentiating DENV and ZIKV infection that can be performed simultaneously in half a day. The reagents or comparable replacements can be easily obtained commercially and easily implemented in laboratories with diagnostic serology experience without the need for expensive or state-of-the-art equipment.

In conclusion, our study demonstrates the development and high performance of an ELISA assay that can discriminate between DENV and ZIKV infections. The implementation of this assay will be important for the diagnostic assessment of travelers to ZIKV and DENV endemic areas, pregnant women and their children who have traveled or live in endemic countries, blood donor screening, and serologic surveys to estimate disease incidence. This assay can aid in studies such as those evaluating neurologic complications associated with ZIKV such as Guillain-Barré syndrome, neuropathy, and myelitis. The differential diagnosis of dengue is also crucial for proper clinical care. Finally, assay implementation must be carefully evaluated and should consider weighing epidemiological factors and molecular evidence of recent ZIKV and DENV circulation.

## Supplemental Material


Supplemental materials
