# Supplementary material for: Capacity of a Multiplex IgM Antibody Capture ELISA to Differentiate Zika and Dengue Virus Infections in Areas of Concurrent Endemic Transmission
Source: Am J Trop Med Hyg. 2021 Dec 20;106(2):585–92. doi: 10.4269/ajtmh.20-1651 (PMC8832915; doi:10.4269/ajtmh.20-1651)
Supplement: Supplementary file 1 [file tpmd201651.SD1.pdf]

Supplemental Table S1a. Testing of challenging DENV specimens in the ZIKV/DENV DUO MAC-ELISA with antigens diluted in PBS containing 137mM or 500mM NaCl.

DENV specimens								
Specimen	PBS (137 mM NaCl)				PBS (500 mM NaCl)			
	DENV antigen OD	ZIKV antigen OD	Z/D Ratio	Test Interpretation	DENV antigen OD	ZIKV antigen OD	Z/D Ratio	Test Interpretation
1	0.186	0.424	2.28	Zika Positive	0.245	0.360	1.47	Equivocal for Dengue
2	0.471	1.286	2.73	Zika Positive	0.476	0.907	1.91	Equivocal for Zika
3	0.424	1.061	2.50	Zika Positive	0.571	0.783	1.37	Dengue Positive
4	0.264	0.955	3.62	Zika Positive	0.280	0.430	1.54	Dengue Positive
5	0.716	0.533	0.74	Dengue Positive	0.680	0.500	0.74	Dengue Positive
6	0.406	0.561	1.38	Dengue Positive	0.488	0.600	1.23	Dengue Positive
7	0.218	0.150	0.69	Dengue Positive	0.272	0.151	0.56	Dengue Positive
8	0.224	0.432	1.93	Equivocal for Zika	0.272	0.266	0.98	Dengue Positive
9	0.513	0.763	1.49	Dengue Positive	0.505	0.605	1.20	Dengue Positive
10	0.157	0.134	0.85	Equivocal for Dengue	0.184	0.163	0.89	Equivocal for Dengue
11	0.253	0.256	1.01	Dengue Positive	0.256	0.278	1.09	Dengue Positive
12	0.138	0.169	1.22	Equivocal for Dengue	0.186	0.207	1.11	Equivocal for Dengue

Note: Challenging specimens were selected based on misclassification or near misclassification during previous testing.

Supplemental Table S1b. Testing of challenging ZIKV specimens in the ZIKV/DENV DUO MAC-ELISA with antigens diluted in PBS containing 137mM or 500mM NaCl.

ZIKV specimens								
	PBS (137 mM NaCl)				PBS (500 mM NaCl)			
Specimen	DENV antigen OD	ZIKV antigen OD	Z/D Ratio	Test Interpretation	DENV antigen OD	ZIKV antigen OD	Z/D Ratio	Test Interpretation
1	0.670	0.519	0.77	Dengue Positive	0.694	0.602	0.87	Dengue Positive
2	0.624	1.126	1.80	Equivocal for Zika	0.574	1.179	2.05	Zika Positive
3	0.334	1.820	5.45	Zika Positive	0.399	1.368	3.43	Zika Positive
4	0.219	0.938	4.28	Zika Positive	0.249	0.613	2.46	Zika Positive
5	0.328	1.158	3.53	Zika Positive	0.379	0.834	2.20	Zika Positive
6	0.375	1.686	4.50	Zika Positive	0.415	1.530	3.69	Zika Positive
7	0.285	1.825	6.40	Zika Positive	0.422	1.861	4.41	Zika Positive
8	0.511	1.059	2.07	Zika Positive	0.488	1.376	2.82	Zika Positive
9	0.164	0.579	3.53	Zika Positive	0.178	0.532	2.99	Zika Positive
10	0.224	1.238	5.53	Zika Positive	0.233	1.761	7.56	Zika Positive
11	0.350	0.874	2.50	Zika Positive	0.372	1.107	2.98	Zika Positive
12	0.167	0.888	5.32	Zika Positive	0.202	0.512	2.53	Zika Positive
13	0.117	0.341	2.91	Zika Positive	0.125	0.295	2.36	Zika Positive
14	0.158	0.442	2.80	Zika Positive	0.183	0.399	2.18	Zika Positive

Note: Challenging specimens were selected based on misclassification or near misclassification during previous testing.

Supplemental Tables S2

ELISA test results (ZIKV IgM ELISA, DENV IgM ELISA, ZIKV DUO IgM ELISA, and DENV DUO IgM ELISA) for DENV, ZIKV, and negative specimens were cross tabulated in order to calculate the proportion of specimens identified correctly using RT-PCR results as a gold standard. Three pairwise comparisons with Bonferroni correction for multiple comparisons were carried out using a T-student test to determine if there were differences in the percent correct results in the ELISA tests according to immune status (ZIKV vs. DENV, ZIKV vs. NEGATIVE, DENV vs. NEGATIVE). Significance was set at 0.05. Statistically significant differences between the scenarios are indicated with an asterisk (*).

Supplemental Table S2a

ZIKV IgM ELISA

	ZIKV IgM ELISA Result				Correct results	
		**ZIKV IgM +**	**ZIKV IgM -**	**Total specimens (N)**	**Number correct**	**% correct**
**PCR result**	ZIKV	103	0	103	103	100.00%
	DENV	81	61	142	61	42.96%
	NEGATIVE	0	143	143	143	100.00%

**Pairwise comparison**	**p-value**
ZIKV vs DENV*	2.68×10^{-20}
ZIKV vs NEGATIVE	NA
DENV vs NEGATIVE*	5.43×10^{-26}

*Significant differences at α=0.001/3 (Bonferroni's correction)

Supplemental Table S2b

DENV IgM ELISA

	DENV IgM ELISA Result				Correct results	
		DENV IgM +	DENV IgM -	Total specimens (N)	Number correct	% correct
PCR result	ZIKV	35	68	103	68	66.02%
	DENV	142	0	142	142	100.00%
	NEGATIVE	0	143	143	143	100.00%

Pairwise comparison	p-value
ZIKV vs DENV*	2.52×10^{-13}
ZIKV vs NEGATIVE*	2.11×10^{-13}
DENV vs NEGATIVE	NA

*Significant differences at α=0.001/3 (Bonferroni's correction)

Supplemental Table S2c

ZIKV DUO IgM ELISA

	ZIKV DUO IgM ELISA result				Correct results	
		ZIKV IgM +	ZIKV IgM -	Total specimens (N)	Number correct	% correct
PCR result	ZIKV	102	1	103	102	99.03%
	DENV	0	142	142	142	100.00%
	NEGATIVE	0	143	143	143	100.00%

Pairwise comparison	p-value
ZIKV vs DENV*	0.8716
ZIKV vs NEGATIVE*	0.8688
DENV vs NEGATIVE	NA

Supplemental Table S2d

DENV DUO IgM ELISA

	DENV DUO IgM ELISA result				Correct results	
		**DENV IgM +**	**DENV IgM -**	**Total specimens (N)**	**Number correct**	**% correct**
**PCR result**	ZIKV	0	0	0	0	NA
	DENV	142	0	142	142	100.00%
	NEGATIVE	0	143	143	143	100.00%

Pairwise comparison	p-value
ZIKV vs DENV	NA
ZIKV vs NEGATIVE	NA
DENV vs NEGATIVE	NA
